# Supplementary material for: Estimating All-Cause Deaths Averted in the First Two Years of the COVID-19 Vaccination Campaign in Italy
Source: Vaccines (Basel). 2024 Apr 13;12(4):413. doi: 10.3390/vaccines12040413 (PMC11055119; doi:10.3390/vaccines12040413)

Estimating all-cause deaths averted in the first two years of the COVID-19 vaccination campaign in Italy

Giovanni Corrao[1,2], Gloria Porcu[1,2,3], Alina Tratsevich[1,2], Danilo Cereda[4],

Giovanni Pavesi[5], Guido Bertolaso[6], Matteo Franchi[1,2]

[1] National Centre for Healthcare Research and Pharmacoepidemiology, University of Milano-Bicocca, Milan, Italy

[2] Unit of Biostatistics, Epidemiology and Public Health, Department of Statistics and Quantitative Methods, University of Milano-Bicocca, Milan, Italy

[3] Specialization School of Health Statistics and Biometrics, University of Padua, Padua, Italy

[4] Preventive Unit of Welfare Department, Lombardy Region, Milan Italy

[5] General Directorate of Welfare Department, Lombardy Region, Milan Italy

[6] Welfare Councillor, Lombardy Region, Milan Italy

Corresponding author:

Dott. Gloria Porcu, Dipartimento di Statistica e Metodi Quantitativi, Università degli Studi di Milano-Bicocca, Via Bicocca degli Arcimboldi, 8, Edificio U7, 20126 Milano, Italy. Tel.: +39.02.64485959; E-mail: gloria.porcu@unimib.it

Annex S1. Localization and age structure (January, 2021) of citizens from the three Italian Regions involved in the current study, compared with the entire Italian population.

**Italy**	
Age categories	
20-49 years	30,569,533 (52.5%)
50-79 years	23,141,534 (39.8%)
≥ 80 years	4,505,526 (7.7%)

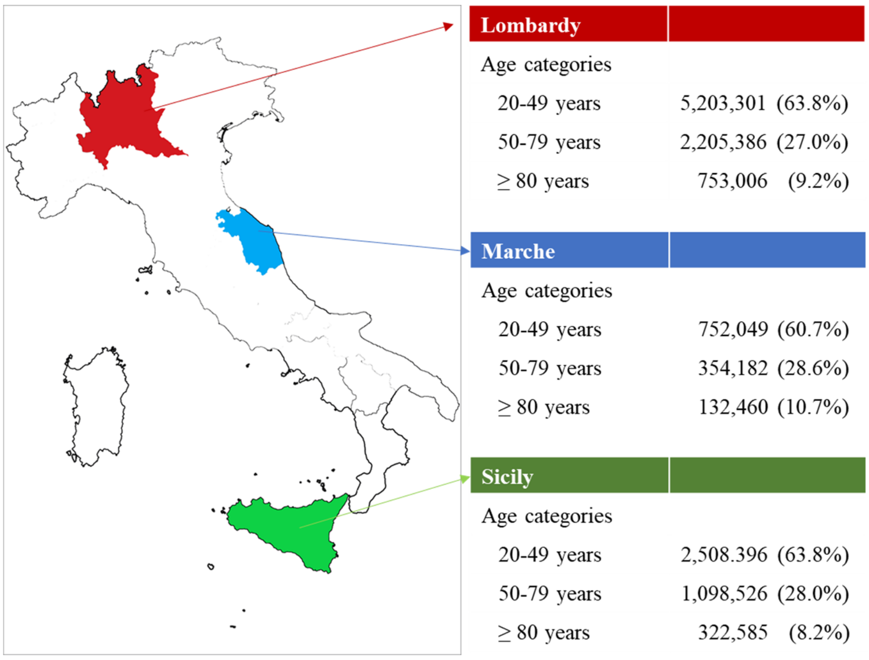

**Lombardy**	
Age categories	
20-49 years	5,203,301 (63.8%)
50-79 years	2,205,386 (27.0%)
≥ 80 years	753,006 (9.2%)

**Marche**	
Age categories	
20-49 years	752,049 (60.7%)
50-79 years	354,182 (28.6%)
≥ 80 years	132,460 (10.7%)

**Sicily**	
Age categories	
20-49 years	2,508.396 (63.8%)
50-79 years	1,098,526 (28.0%)
≥ 80 years	322,585 (8.2%)

Annex S2. Seasonal autoregressive integrated moving average with exogenous factors (SARIMAX) modelling excess mortality.

Excess mortality was defined as the difference between the observed and benchmark death counts. While the observed mortality was the actual death count during the vaccinal period (i.e., from December 2020 until October 2022), the benchmark mortality was defined as the estimated death count according to historical trends, excluding the influence of COVID-19 (i.e., during the years 2015-2019). In other words, the expected death count during the vaccinal period provided that the mortality process exhibited a "natural variability" as that of the no-COVID period (the benchmark based on the counterfactual condition of COVID-19).

The ARIMA (autoregressive integrated moving average) model, which uses auto-regression and moving average, and incorporates a differencing order to remove trend and/or seasonality [1], is one of the most widely used statistical methods for forecasting stationary time series [2].

Particularly, because the time process of interest (daily count of all-cause deaths) forms a time series with seasonal variations, an extension of ARIMA, the so-called SARIMA (seasonal ARIMA) was used in this study [3-7]. In addition, besides seasons, other exogenous factors likely affect the time process of interest (e.g., the environmental temperature); therefore, a SARIMAX (seasonal autoregressive integrated moving average with exogenous factors) model was fitted for estimating the expected death count (X_t). SARIMAX assumes the following form:

$$X_t = \beta_0 + \beta_0 X_t^{(1)} + m_t + s_t + Y_t$$

where β_0 indicates the intercept and $\beta_0 X_t^{(1)}$, m_t, s_t, and Y_t the effects of exogenous factors (in our case, the temperature at time t), trend, season, and noise, respectively.

The ARIMA model contains 3 parameters (p, d, q): (i) parameter p represents the periods to lag for; (ii) parameter d represents the number of differencing transformations done to remove trend and/or seasonality therefore turning the time-series into a stationary one, i.e, making the mean and variance constant over time; (iii) parameter q represents the lag of the error component of the ARIMA model, which is the part of the time-series that cannot be explained by trend or seasonality. Selecting appropriate values for parameters p and q requires testing and optimization. To choose the values, one must inspect visual observations of the data to determine trend and/or seasonality in the form of auto-correlation function (ACF) and partial auto-correlation function (PACF) charts [1].

In our study, the input variables were measured during the control period from January 1, 2015, to December 31, 2019, and included the time series of daily observed deaths reported by the Italian National Institute of Statistics [8] stratified by Italian region and age category, and the daily temperatures tracked by the Italian National Environmental Protection System official site [9].

Selection of the parameters to be set in the SARIMAX model was performed by using the "auto.arima" package of the 4.0.2 version of the R statistical software, which returns the best results based on the values of the Akaike's Information Criterion (AIC), corrected AIC (AICc) or Bayesian Information Criteria (BIC). Specifically, using the "auto.arima" package, these values were used to compare the models in terms of quality, which is achieved by finding a balance between the fit of the model and its complexity [1].

Annex S3. **Modelling the prevented fraction.**

Notations

d — generic day of the vaccinal period, with d=0,….,d=D; that is, from February 1, 2021 (14 days after the first Italian citizen completed the first COVID-19 vaccine dose) to October 31, 2022 (the last date with available data for the current paper)

r — Italian region, with r = 1, 2, and 3 for citizens from Lombardy, Marche, and Sicily, respectively

a — age category, with a = 1, 2, and 3 for citizens aged 20-49, 50-79, and 80 years or older, respectively

v — vaccine dose, with v = 1, 2, or 3 indicating the first complete dose, and the first and second booster doses, respectively

j — days elapsed since receiving the last vaccine dose, with j=1,…,J

s — viral original (v=0) or Omicron (v=1) variant of SARS-CoV-2

Remarks

The cumulative prevented fraction (PF) from February 1, 2021, was calculated from:

$$PF_{rad} = \sum_{v=1}^{3}\sum_{j=1}^{J} PV_{ravd} \times VE_{avjd}$$

The first quantity, PV_{ravd}, indicates the cumulative prevalence of citizens resident in each region, belonging to a given age category, reached by a given dose of vaccine, each day d of the vaccinal period. Under the assumption that clinically significant immunity is achieved two weeks after receiving the vaccine [10], we moved the effective date of vaccine administration for counting the daily number of vaccinated forward by 14 days. Because PV_{ra1d}, PV_{ra2d}, and PV_{ra3d} included citizens who received the (i) first dose (but not the second and third ones), (ii) second dose (but not the third one), and (iii) third dose, respectively, exposure to one, two, or three doses is to be considered mutually exclusive and independent.

The second quantity, VE_{avjs}, indicates the estimated vaccine effectiveness (VE) in avoiding deaths. VE is assumed to be invariant between regions, but heterogeneous according with age, dose, time elapsed since vaccination, and prevalent viral strain. To model VE according to these parameters, we proceeded as follows:

First, a systematic review and meta-analysis was conducted to summarize meta-analytic estimates of VE according to age and dose. We searched PubMed to November 15, 2022, for observational studies reporting estimates of VE against death, stratified by age and vaccine dose, of the two mRNA-based (manufactured by Pfizer and Moderna) and two adenovirus-vectored (manufactured by Oxford-AstraZeneca and Janssen) vaccines available in Italy. Out of 961 original papers and 7 systematic reviews, 19 research articles were included in the final meta-analysis [11-29]. Among these, 17, 5, and 3 reported age-specific VE estimates for the first, second, and third dose, respectively. The following table shows the random effect meta-analytic estimates of VE among strata of age and vaccine dose:

	Meta-analytic (pooled) estimate of VE and 95% Confidence Interval	Number of studies considered
Age 20-49 years		
1 dose	87% (81% - 90%)	3
2 doses	Not available	0
3 doses	Not available	0
Age 50-79 years		
1 dose	94% (91% - 95%)	19
2 doses	96% (93% - 98%)	4
3 doses	91% (66% - 98%)	2
Age 80+ years		
1 dose	80% (79% - 82%)	20
2 doses	86% (74% - 92%)	4
3 doses	81% (76% - 86%)	3

Since, to the best of our knowledge, estimates of the second and third dose effects in people aged less than 50 years are not available, the summarized VE for people aged 50 to 79 years (that is, 96% and 91%) were used.

Second, with the aim of considering the waning of VE over time, we considered data from a published population-based study conducted on more than 9 million beneficiaries of the Lombardy Regional Health Service [30], having the same setting of one of the 3 regions considered in the current study. Particularly, we extracted the monthly VE values against severe COVID-19 illness, and we evaluated the decrease over time. Starting from these data, we fitted a second-degree polynomial function to predict the daily VE decline from 14 days to nine months after vaccine administration. The polynomial function assumed the following form: $VE_j = 1 - (0.186 - 0.00151 \times j + 0.000014 \times j^2)$.

Finally, to account for the reduced effectiveness of mRNA and viral-vector vaccines in epidemic periods led by different SARS-CoV-2 variants, summarized estimates from four systematic reviews of the literature on this topic [31-34] and two additional original papers [35,36], lead of considering decreased VE values of 5% starting from January 2022, the month when the Omicron variant became dominant in Italy [37].

Finally, the overall cumulative prevented fraction in each region according to the vaccination speed in reaching citizens with one, two, and three vaccine doses was calculated, net of between-region differences in age structure, by applying the direct standardization:

$$FP_r = \sum_{v=1}^{3} \sum_{a=1}^{3} FP_{ra} \times p'_a$$

where p'_a indicates the proportion of Italian citizens belonging to a given age category, with respect to Italian citizens aged 20 years or older (Supplemental Material, **Annex S1**, reports the corresponding values).

References

[1] Alabdulrazzaq H, Alenezi MN, Rawajfih Y, Alghannam BA, Al-Hassan AA, Al-Anzi FS. On the accuracy of ARIMA based prediction of COVID-19 spread. Results Phys. 2021 Aug;27:104509. doi: 10.1016/j.rinp.2021.104509. Epub 2021 Jul 15. PMID: 34307005; PMCID: PMC8279942.

[2] Box, George E. P., and Gwilym M. Jenkins. 1976. Time Series Analysis: Forecasting and Control. San Francisco: Holden-Day.

[3] ArunKumar, K. E., Dinesh V. Kalaga, Ch. Mohan S. Kumar, Govinda Chilkoor, Masahiro Kawaji, and Timothy M. Brenza. 2021. Forecasting the dynamics of cumulative COVID-19 cases (confirmed, recovered and deaths) for top-16 countries using statistical machine learning models: Auto-Regressive Integrated Moving Average (ARIMA) and Seasonal Auto-Regressive Integrated Moving Average (SARIMA). Applied Soft Computing 103: 107161

[4] Cong, Jing, Mengmeng Ren, Shuyang Xie, and Pingyu Wang. 2019. Predicting Seasonal Influenza Based on SARIMA Model, in Mainland China from 2005 to 2018. International Journal of Environmental Research and Public Health 16: 4760

[5] Hossain MS, Ahmed S, Uddin MJ. Impact of weather on COVID-19 transmission in south Asian countries: An application of the ARIMAX model. Sci Total Environ. 2021 Mar 20;761:143315.

[6] Du M, Zhu H, Yin X, Ke T, Gu Y, Li S, Li Y, Zheng G. Exploration of influenza incidence prediction model based on meteorological factors in Lanzhou, China, 2014-2017. PLoS One. 2022 Dec 15;17(12):e0277045. doi: 10.1371/journal.pone.0277045. PMID: 36520836; PMCID: PMC9754291.

[7] Du M, Zhu H, Yin X, Ke T, Gu Y, Li S, Li Y, Zheng G. Exploration of influenza incidence prediction model based on meteorological factors in Lanzhou, China, 2014-2017. PLoS One. 2022 Dec 15;17(12):e0277045. doi: 10.1371/journal.pone.0277045. PMID: 36520836; PMCID: PMC9754291.

[8] National Institute of Statistics. Mortality data. Available at: Available at: https://www.istat.it/en/archive/deaths (last checked April 05, 2023)

[9] Sistema Nazionale per la Protezione dell'Ambiente. Available at: www.snpambiente.it/chi-siamo/i-nodi-del-sistema/i-siti-web (last checked April 05, 2023)

[10] Dagan N, Barda N, Kepten E, et al. BNT162b2 mRNA Covid-19 vaccine in a nationwide mass vaccination setting. N Engl J Med 2021; 384: 1412–23

[11] Lopez Bernal J, Andrews N, Gower C, et al. Effectiveness of the Pfizer-BioNTech and Oxford-AstraZeneca vaccines on covid-19 related symptoms, hospital admissions, and mortality in older adults in England: test negative case-control study. BMJ. 2021 May 13;373:n1088.

[12] Alencar CH, Cavalcanti LPG, Almeida MM, et al. High Effectiveness of SARS-CoV-2 Vaccines in Reducing COVID-19-Related Deaths in over 75-Year-Olds, Ceará State, Brazil. Trop Med Infect Dis. 2021 Jul 13;6(3):129.

[13] Arbel R, Sergienko R, Friger M, et al. Effectiveness of a second BNT162b2 booster vaccine against hospitalization and death from COVID-19 in adults aged over 60 years. Nat Med. 2022 Jul;28(7):1486-1490.

[14] Anderegg N, Althaus CL, Colin S,et al. Assessing real-world vaccine effectiveness against severe forms of SARS-

CoV-2 infection: an observational study from routine surveillance data in Switzerland. Swiss Med Wkly. 2022 Apr 19;152:w30163.

[15] Arregocés-Castillo L, Fernández-Niño J, Rojas-Botero M, et al. Effectiveness of COVID-19 vaccines in older adults in Colombia: a retrospective, population-based study of the ESPERANZA cohort. Lancet Healthy Longev. 2022 Apr;3(4):e242-e252. Erratum in: Lancet Healthy Longev. 2022 Aug;3(8):e518.

[16] Kislaya I, Machado A, Magalhães S, et al. COVID-19 mRNA vaccine effectiveness (second and first booster dose) against hospitalisation and death during Omicron BA.5 circulation: cohort study based on electronic health records, Portugal, May to July 2022. Euro Surveill. 2022 Sep;27(37):2200697.

[17] Glatman-Freedman A, Bromberg M, Dichtiar R, et al. The BNT162b2 vaccine effectiveness against new COVID-19 cases and complications of breakthrough cases: A nation-wide retrospective longitudinal multiple cohort analysis using individualised data. EBioMedicine. 2021 Oct;72:103574.

[18] Haas EJ, Angulo FJ, McLaughlin JM, et al. Impact and effectiveness of mRNA BNT162b2 vaccine against SARS-CoV-2 infections and COVID-19 cases, hospitalisations, and deaths following a nationwide vaccination campaign in Israel: an observational study using national surveillance data. Lancet. 2021 May 15;397(10287):1819-1829. Erratum in: Lancet. 2021 Jul 17;398(10296):212.

[19] Goldberg Y, Mandel M, Woodbridge Y, et al. Similarity of Protection Conferred by Previous SARS-CoV-2 Infection and by BNT162b2 Vaccine: A 3-Month Nationwide Experience From Israel. Am J Epidemiol. 2022 Jul 23;191(8):1420-1428.

[20] Kiss Z, Wittmann I, Polivka L, et al. Nationwide Effectiveness of First and Second SARS-CoV2 Booster Vaccines During the Delta and Omicron Pandemic Waves in Hungary (HUN-VE 2 Study). Front Immunol. 2022 Jun 23;13:905585.

[21] Nunes B, Rodrigues AP, Kislaya I, et al. mRNA vaccine effectiveness against COVID-19-related hospitalisations and deaths in older adults: a cohort study based on data linkage of national health registries in Portugal, February to August 2021. Euro Surveill. 2021 Sep;26(38):2100833.

[22] Lytras T, Kontopidou F, Lambrou A, et al. Comparative effectiveness and durability of COVID-19 vaccination against death and severe disease in an ongoing nationwide mass vaccination campaign. J Med Virol. 2022 Oct;94(10):5044-5050.

[23] McMenamin ME, Nealon J, Lin Y, Wet al. Vaccine effectiveness of one, two, and three doses of BNT162b2 and CoronaVac against COVID-19 in Hong Kong: a population-based observational study. Lancet Infect Dis. 2022 Oct;22(10):1435-1443. Erratum in: Lancet Infect Dis. 2022 Sep;22(9):e239.

[24] Nordström P, Ballin M, Nordström A. Risk of infection, hospitalisation, and death up to 9 months after a second dose of COVID-19 vaccine: a retrospective, total population cohort study in Sweden. Lancet. 2022 Feb 26;399(10327):814-823.

[25] Saciuk Y, Kertes J, Mandel M, et al. Pfizer-BioNTech vaccine effectiveness against Sars-Cov-2 infection: Findings from a large observational study in Israel. Prev Med. 2022 Feb;155:106947.

[26] Wan EYF, Mok AHY, Yan VKC, et al. Effectiveness of BNT162b2 and CoronaVac vaccinations against SARS-CoV-2 omicron infection in people aged 60 years or above: a case-control study. J Travel Med. 2022 Dec 27;29(8):taac119.

[27] Sheikh A, Robertson C, Taylor B. BNT162b2 and ChAdOx1 nCoV-19 Vaccine Effectiveness against Death from the Delta Variant. N Engl J Med. 2021 Dec 2;385(23):2195-2197.

[28] Vokó Z, Kiss Z, Surján Get al. Nationwide effectiveness of five SARS-CoV-2 vaccines in Hungary-the HUN-

VE study. Clin Microbiol Infect. 2022 Mar;28(3):398-404.
[29] Emborg HD, Valentiner-Branth P, Schelde AB et al. Vaccine effectiveness of the BNT162b2 mRNA COVID-19 vaccine against RT-PCR confirmed SARS-CoV-2 infections, hospitalisations and mortality in prioritised risk groups. medRxiv 2021.05.27.21257583; doi: https://doi.org/10.1101/2021.05.27.21257583
[30] Corrao G, Franchi M, Cereda D, et al. Persistence of protection against SARS-CoV-2 clinical outcomes up to 9 months since vaccine completion: a retrospective observational analysis in Lombardy, Italy. Lancet Infect Dis 2022;22:649-56
[31] Zhang J, Yang W, Huang F, et al. Effectiveness of mRNA and viral-vector vaccines in epidemic period led by different SARS-CoV-2 variants: a systematic review and meta-analysis. J Med Virol 2023 Feb 28. doi: 10.1002/jmv.28623. Epub ahead of print. PMID: 36852651
[32] Shao W, Chen X, Zheng C, et al. Effectiveness of COVID-19 vaccines against SARS-CoV-2 variants of concern in real-world: a literature review and meta-analysis. Emerg Microbes Infect 2022;11:2383-92
[33] Wang K, Wang L, Li M, et al. Real-Word Effectiveness of Global COVID-19 Vaccines Against SARS-CoV-2 Variants: A Systematic Review and Meta-Analysis. Front Med (Lausanne) 2022;9:820544
[34] Zeng B, Gao L, Zhou Q, et al. Effectiveness of COVID-19 vaccines against SARS-CoV-2 variants of concern: a systematic review and meta-analysis. BMC Med 2022;20:200
[35] Buchan SA, Chung H, Brown KA, et al. Estimated Effectiveness of COVID-19 Vaccines Against Omicron or Delta Symptomatic Infection and Severe Outcomes. JAMA Netw Open. 2022 Sep 1;5(9):e2232760.
[36] Gram MA, Emborg HD, Schelde AB, et al. Vaccine effectiveness against SARS-CoV-2 infection or COVID-19 hospitalization with the Alpha, Delta, or Omicron SARS-CoV-2 variant: A nationwide Danish cohort study. PLoS Med. 2022 Sep 1;19(9):e1003992.
[37] Stefanelli P, Trentini F, Petrone D, et al; Tracking the progressive spread of the SARS-CoV-2 Omicron variant in Italy, December 2021 to January 2022. Euro Surveill. 2022 Nov;27(45):2200125.

Annex S4. **Sensitivity analyses (methods).**

Sensitivity analysis 1

The following values of vaccine effectiveness (VE) specific for each age category and vaccinal dose was assumed as an alternative to those used for the main analysis (**Annex S3**):

	Meta-analytic (summarized) estimate of VE
Age 20-49 years	
1 dose	87%
2 doses	96%
3 doses	96%
Age 50-79 years	
1 dose	94%
2 doses	96%
3 doses	96%
Age 80+ years	
1 dose	80%
2 doses	86%
3 doses	86%

VE: vaccine effectiveness

Sensitivity analysis 2

A 1% decrease in VE starting from January 2022 (i.e., when the Omicron variant became dominant in Italy) was assumed instead of 5% as in the main analysis.

Sensitivity analysis 3

Values of VE specific for each age category and vaccinal dose as in sensitivity analysis 1 and a 1% decrease in VE starting from January 2022 (when Omicron variant became dominant in Italy) was assumed instead of 5% as in the main analysis.

Annex S5. Number of positive swabs (Supplementary Table S1) and number of vaccination doses inoculated over time (Supplementary Table S2) in the three Regions**.**

Supplementary Table S1

Period	Lombardy	Marche	Sicily
December 2020	6,948	1,927	4,028
January 2021	59,151	13,863	42,459
February 2021	65,027	12,370	16,455
March 2021	132,433	20,289	21,565
April 2021	69,136	9,559	34,364
May 2021	30,497	4,977	17,322
June 2021	6,708	958	5,887
July 2021	11,145	1,922	11,244
August 2021	16,724	4,955	33,590
September 2021	14,050	3,340	21,530
October 2021	10,839	2,375	9,856
November 2021	37,961	7,627	15,503
December 2021	284,790	21,605	48,146
January 2022	911,717	115,447	249,480
February 2022	203,479	66,254	152,679
March 2022	204,667	63,411	150,579
April 2022	228,126	54,014	118,791
May 2022	127,540	28,936	68,678
June 2022	158,400	25,621	85,771
July 2022	319,875	76,225	184,424
August 2022	102,225	29,386	61,862
September 2022	100,108	18,232	29,465
October 2022	209,057	31,082	38,363

Supplementary Table S2

Period	Lombardy				Marche				Sicily			
	No vaccine	1 dose	2 doses	3 doses	No vaccine	1 dose	2 doses	3 doses	No vaccine	1 dose	2 doses	3 doses
December 2020	100.0%	0.0%	0.0%	0.0%	100.0%	0.0%	0.0%	0.0%	100.0%	0.0%	0.0%	0.0%
January 2021	99.1%	0.9%	0.0%	0.0%	98.8%	1.2%	0.0%	0.0%	98.4%	1.6%	0.0%	0.0%
February 2021	96.9%	3.1%	0.0%	0.0%	97.6%	2.4%	0.0%	0.0%	97.2%	2.8%	0.0%	0.0%
March 2021	93.5%	6.5%	0.0%	0.0%	92.0%	8.0%	0.0%	0.0%	94.0%	6.0%	0.0%	0.0%
April 2021	88.1%	11.9%	0.0%	0.0%	87.6%	12.4%	0.0%	0.0%	88.7%	11.3%	0.0%	0.0%
May 2021	75.0%	25.0%	0.0%	0.0%	75.2%	24.8%	0.0%	0.0%	77.1%	22.9%	0.0%	0.0%
June 2021	65.3%	34.7%	0.0%	0.0%	64.6%	35.4%	0.0%	0.0%	65.7%	34.3%	0.0%	0.0%
July 2021	36.5%	63.5%	0.0%	0.0%	38.4%	61.6%	0.0%	0.0%	44.0%	56.0%	0.0%	0.0%
August 2021	27.2%	72.8%	0.0%	0.0%	30.5%	69.5%	0.0%	0.0%	36.9%	63.1%	0.0%	0.0%
September 2021	20.3%	79.5%	0.2%	0.0%	24.5%	75.3%	0.2%	0.0%	29.7%	70.2%	0.1%	0.0%
October 2021	17.2%	79.5%	3.4%	0.0%	21.2%	76.1%	2.7%	0.0%	25.6%	73.0%	1.4%	0.0%
November 2021	15.9%	69.9%	14.1%	0.0%	19.4%	66.7%	14.0%	0.0%	23.3%	67.6%	9.1%	0.0%
December 2021	15.3%	40.6%	44.1%	0.0%	18.5%	41.8%	39.8%	0.0%	22.0%	49.4%	28.6%	0.0%
January 2022	14.5%	14.4%	71.1%	0.0%	17.3%	16.4%	66.3%	0.0%	20.2%	24.5%	55.4%	0.0%
February 2022	13.6%	7.8%	78.6%	0.0%	16.3%	11.6%	72.1%	0.0%	17.9%	18.8%	63.3%	0.0%
March 2022	13.4%	5.5%	81.0%	0.1%	15.9%	9.7%	74.3%	0.1%	17.0%	16.8%	66.1%	0.0%
April 2022	13.3%	4.5%	81.4%	0.8%	15.8%	8.9%	74.8%	0.5%	16.9%	16.0%	66.9%	0.2%
May 2022	13.3%	4.0%	80.6%	2.1%	15.8%	8.7%	74.4%	1.1%	16.8%	15.6%	66.9%	0.6%
June 2022	13.3%	3.5%	80.5%	2.7%	15.8%	8.5%	74.3%	1.4%	16.8%	15.4%	66.9%	0.9%
July 2022	13.3%	3.0%	78.2%	5.5%	15.8%	8.2%	72.9%	3.0%	16.8%	15.1%	66.0%	2.2%
August 2022	13.3%	2.8%	76.8%	7.1%	15.8%	8.1%	71.9%	4.3%	16.8%	14.9%	65.7%	2.6%
September 2022	13.3%	2.7%	75.9%	8.2%	15.8%	8.0%	71.4%	4.8%	16.7%	14.8%	65.5%	2.9%
October 2022	13.3%	2.5%	72.4%	11.9%	15.7%	7.9%	69.4%	7.0%	16.7%	14.7%	64.5%	4.0%

Annex S6. **Sensitivity analyses (results).**

Sensitivity analysis 1

Different values of vaccine effectiveness (VE) specific for each age category and vaccinal dose was assumed as an alternative to those used for the main analysis.

Supplementary Figure S1. Daily time series of the prevented fraction from January 01, 2021, to October 31, 2022, in the Italian regions of Lombardy, Marche, and Sicily.

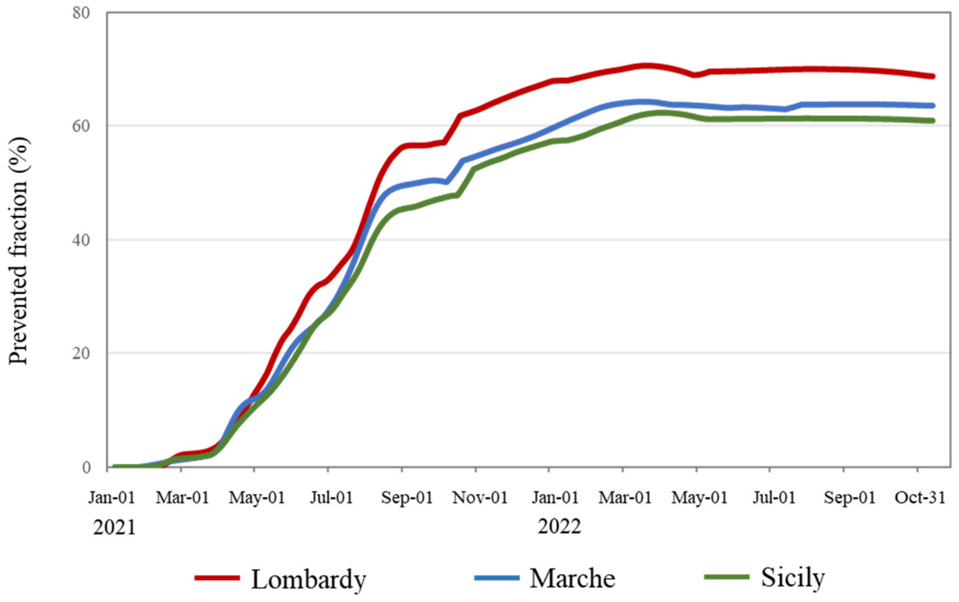

Supplementary Figure S2. Daily time series of the cumulative number of excess deaths (i) observed (net of seasonality and temperature); (ii) avoided; and (iii) would have occurred without vaccination (obtained by considering the first and second quantities together) in the Italian regions of Lombardy, Marche, and Sicily.

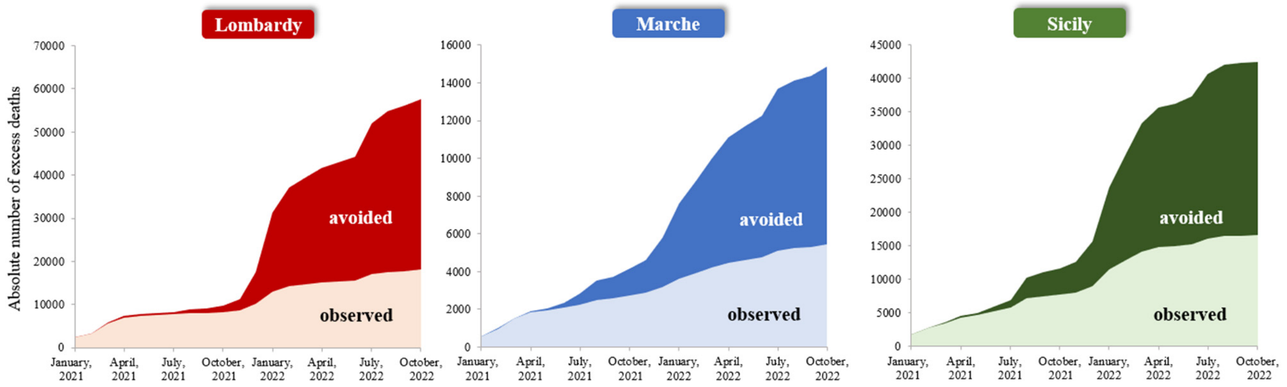

Sensitivity analysis 2

A 1% decrease in VE starting from January 2022 (i.e., when the Omicron variant became dominant in Italy) was assumed instead of 5% as in the main analysis.

Supplementary Figure S3. Daily time series of the prevented fraction from January 01, 2021, to October 31, 2022, in the Italian regions of Lombardy, Marche, and Sicily.

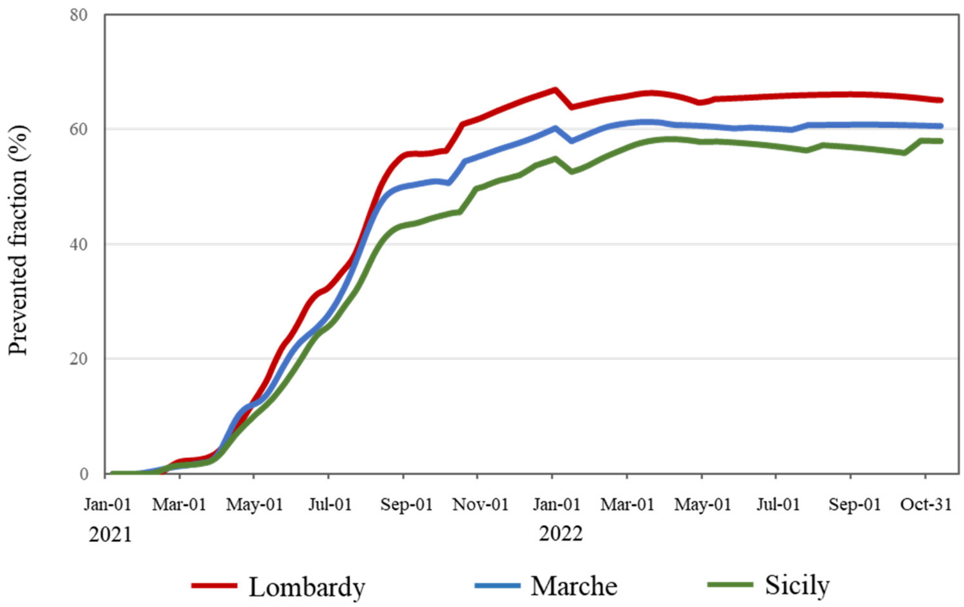

Supplementary Figure S4. Daily time series of the cumulative number of excess deaths (i) observed (net of seasonality and temperature); (ii) avoided; and (iii) would have occurred without vaccination (obtained by considering the first and second quantities together) in the Italian regions of Lombardy, Marche, and Sicily.

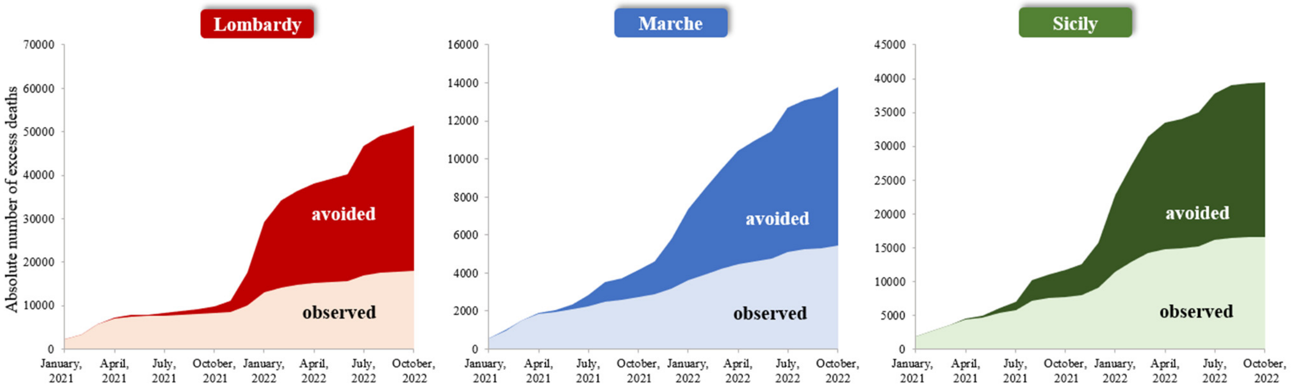

Sensitivity analysis 3

Values of VE specific for each age category and vaccinal dose as in sensitivity analysis 1 and a 1% decrease in VE starting from January 2022 (when Omicron variant became dominant in Italy) was assumed instead of 5% as in the main analysis.

Supplementary Figure S5. Daily time series of the prevented fraction from January 01, 2021, to October 31, 2022, in the Italian regions of Lombardy, Marche, and Sicily.

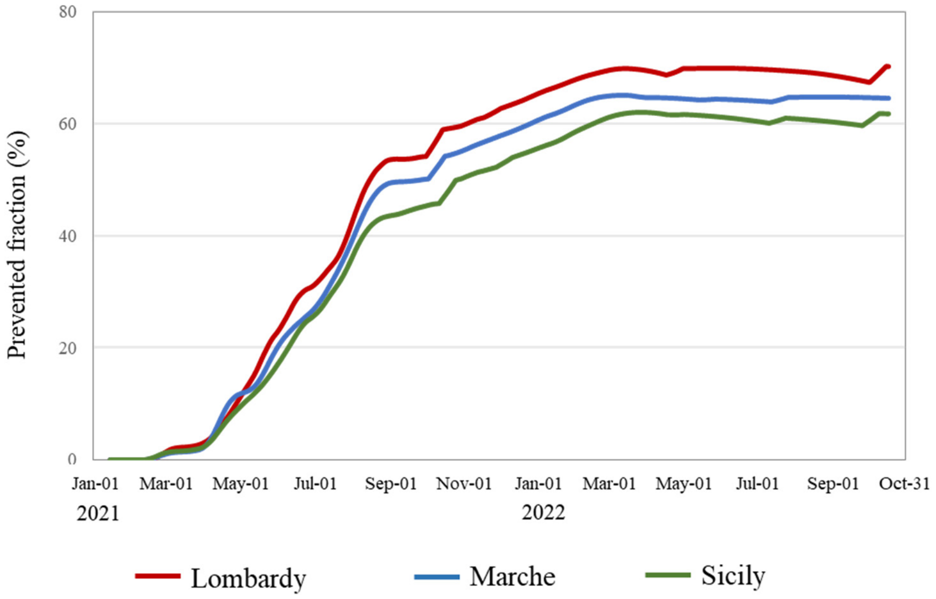

Supplementary Figure S6. Daily time series of the cumulative number of excess deaths (i) observed (net of seasonality and temperature); (ii) avoided; and (iii) would have occurred without vaccination (obtained by considering the first and second quantities together) in the Italian regions of Lombardy, Marche, and Sicily.

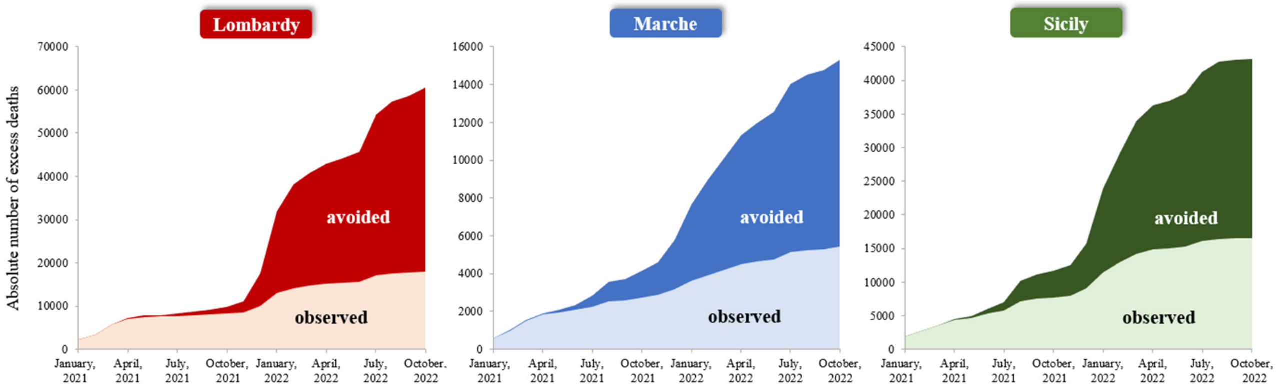

Supplement: Supplementary file 1 [file vaccines-12-00413-s001.zip › vaccines-2918802-supplementary.pdf]
